# Crystal Structure and Functional Analysis of the SARS-Coronavirus RNA Cap 2′-O-Methyltransferase nsp10/nsp16 Complex

**DOI:** 10.1371/journal.ppat.1002059

**Published:** 2011-05-26

**Authors:** Etienne Decroly, Claire Debarnot, François Ferron, Mickael Bouvet, Bruno Coutard, Isabelle Imbert, Laure Gluais, Nicolas Papageorgiou, Andrew Sharff, Gérard Bricogne, Miguel Ortiz-Lombardia, Julien Lescar, Bruno Canard

**Affiliations:** 1 Centre National de la Recherche Scientifique and Université de la Méditerranée, UMR 6098, Architecture et Fonction des Macromolécules Biologiques, Marseille, France; 2 Global Phasing Ltd., Sheraton House, Castle Park, Cambridge, United Kingdom; 3 School of Biological Sciences, Nanyang Technological University, Singapore, Republic of Singapore; Institut Pasteur, France

## Abstract

Cellular and viral S-adenosylmethionine-dependent methyltransferases are involved in many regulated processes such as metabolism, detoxification, signal transduction, chromatin remodeling, nucleic acid processing, and mRNA capping. The Severe Acute Respiratory Syndrome coronavirus nsp16 protein is a S-adenosylmethionine-dependent (nucleoside-2′-O)-methyltransferase only active in the presence of its activating partner nsp10. We report the nsp10/nsp16 complex structure at 2.0 Å resolution, which shows nsp10 bound to nsp16 through a ∼930 Å^2^ surface area in nsp10. Functional assays identify key residues involved in nsp10/nsp16 association, and in RNA binding or catalysis, the latter likely through a SN2-like mechanism. We present two other crystal structures, the inhibitor Sinefungin bound in the S-adenosylmethionine binding pocket and the tighter complex nsp10(Y96F)/nsp16, providing the first structural insight into the regulation of RNA capping enzymes in (+)RNA viruses.

## Author Summary

A novel coronavirus emerged in 2003 and was identified as the etiological agent of the deadly disease called Severe Acute Respiratory Syndrome. This coronavirus replicates and transcribes its giant genome using sixteen non-structural proteins (nsp1-16). Viral RNAs are capped to ensure stability, efficient translation, and evading the innate immunity system of the host cell. The nsp16 protein is a RNA cap modifying enzyme only active in the presence of its activating partner nsp10. We have crystallized the nsp10/16 complex and report its crystal structure at atomic resolution. Nsp10 binds to nsp16 through a ∼930 Å^2^ activation surface area in nsp10, and the resulting complex exhibits RNA cap (nucleoside-2′-O)-methyltransferase activity. We have performed mutational and functional assays to identify key residues involved in catalysis and/or in RNA binding, and in the association of nsp10 to nsp16. We present two additional crystal structures, that of the known inhibitor Sinefungin bound in the SAM binding pocket, and that of a tighter complex made of the mutant nsp10(Y96F) bound to nsp16. Our study provides a basis for antiviral drug design as well as the first structural insight into the regulation of RNA capping enzymes in (+)RNA viruses.

## Introduction

Most eukaryotic cellular and viral mRNAs are modified by the addition of a polyadenine tail at the 3′- terminal and a cap structure at the 5′-terminal. The RNA cap protects mRNA from degradation by 5′ exoribonucleases, ensures efficient mRNA translation, and prevents recognition of viral RNA *via* innate immunity mechanisms[1], [2], [3], [4]. The RNA cap is made of an N7-methylated guanine nucleotide connected through a 5′-5′ triphosphate bridge to the first transcribed nucleotide, generally an adenine. Through 2′-O methylation of the latter, this cap-0 structure (^7Me^GpppA…) may be converted into a cap-1 structure (^7Me^GpppA_2′-O-Me_…). In the eukaryotic cell, the cap is added co-transcriptionally in the nucleus by three sequential enzymatic reactions[1], [5]: (i) an RNA triphosphatase (RTPase) removes the 5′ γ-phosphate group of the nascent mRNA; (ii) a guanylyltransferase (GTase), dubbed capping enzyme, catalyses the attachment of GMP to the 5′-diphosphate mRNA; and (iii) an S-adenosylmethionine (SAM)-dependent (N7-guanine)-methyltransferase (N7MTase) methylates the cap onto the N7-guanine, releasing S-adenosylhomocysteine (SAH). In general, a SAM-dependent (nucleoside-2′-O-)-methyltransferase (2′-O-MTase) further intervenes, in higher eukaryotes, to yield a cap-1 structure.

The viral RNA capping machinery is structurally and mechanistically diverse, and RNA viruses often deviate from the paradigmic eukaryotic mRNA capping scheme. For example, alphaviruses methylate GTP onto the N7-guanine before the presumed attachment of ^7Me^GMP to the nascent viral 5′-diphosphate mRNA[6]. In the case of single-stranded negative-sense (-)RNA viruses, such as the vesicular stomatitis virus, the L polymerase attaches GDP rather than GMP to a nascent viral 5′-monophosphate mRNA, covalently linked to the viral capping enzyme[7]. Other viruses, such as influenza virus capture a short capped RNA oligonucleotide from host cell mRNAs and use it as an RNA synthesis primer. This process is known as « cap snatching »[8].

In 2003, a novel coronavirus named Severe Acute Respiratory Syndrome coronavirus (SARS-CoV[9]) was responsible for the first viral pandemic of the new millennium with ∼8000 cases globally and a 10 % case-fatality rate. Coronaviruses encode an unusually large membrane-associated RNA replication/transcription machinery comprising at least sixteen proteins (nsp1-to-16)[10]. For SARS-CoV, the RNA cap structure likely corresponds to a cap-1 type[11], [12], [13]. As in many other (+)RNA viruses, the RTPase activity is presumably embedded in the RNA helicase nsp13, whereas the GTase remains elusive. RNA cap 2′-O-MTase activity was first discovered in the feline coronavirus (FCoV) nsp16[14]. Shortly after, SARS-CoV nsp14 was shown to methylate RNA caps in their N7-guanine position[15]. Curiously, although closely homologous to that of FCoV, recombinant SARS-CoV nsp16 alone was devoid of enzymatic activity. It was demonstrated[16], [17], [18], [19] that nsp10 interacts with nsp16, conferring 2′-O-MTase activity to nsp16 on N7-methyl guanine RNA caps selectively[16]. The latter selectivity implies that RNA cap methylation obeys an ordered sequence of events during which nsp14-mediated N7-guanine methylation precedes nsp10/nsp16 RNA 2′-O methylation. Nsp10 is a double zinc finger protein of 148 residues whose crystal structure is known[20], [21]. Together with nsp4, nsp5, nsp12, nsp14, and nsp16, nsp10 has been found to be essential in the assembly of a functional replication/transcription complex[22]. Drawing on these observations, nsp10 has been proposed to play pleiotropic roles in viral RNA synthesis[23] and polyprotein processing through interaction with the main protease nsp5[24].

SAM-dependent MTases belong to a large class of enzymes present in all life forms. These enzymes catalyze the transfer of the SAM methyl group to a wide spectrum of methyl acceptors, indicating that a common chemical reaction is used on a variable active-site environment able to activate the methyl acceptor atom. Although SAM-dependent MTases share little sequence identity, 2′O-MTases exhibit a KDKE catalytic tetrad and a very conserved folding made of a seven-stranded β-sheet surrounded by one to three helices on each side[25], always similar to the paradigmatic catechol-O-MTase[26]. The SAM binding site general location is conserved, suggesting that evolutionary pressure on the MTase fold has maintained the same SAM-binding region whilst accommodating the versatile chemistry of the methyltransfer reaction.

Structural and functional studies of viral MTases involved in RNA capping is an expanding research area, since these enzymes show unexpected diversity relative to their cellular counterparts, and thus constitute attractive antiviral targets. Crystal structures of viral RNA cap MTases exist for only three viral families, namely *Poxviridae*, *Reoviridae*, and *Flaviviridae*. The Vaccinia virus VP39 crystal structure was the first to be elucidated in 1996[27]. The structure of this DNA virus RNA 2′-O-MTase revealed a conserved MTase fold similar to that of RrmJ (also named FtsJ), the canonical reference folding for RNA cap MTases[26]. More recently, the crystal structure of a second Vaccinia virus N7-guanine RNA cap MTase domain (D1) was determined in complex with its activator protein D12[28]. The study revealed that D12 also bears an MTase fold, but has lost catalytic capability due to truncation of its SAM binding site. In turn, *Reoviridae* provided the first RNA cap MTase structures at 3.6 Å resolution as forming part of the reovirus core[29]. Another RNA cap machinery was more recently described for the non-turreted orbivirus Bluetongue virus VP4 protein at 2.5 Å resolution[30], which revealed a three-domain protein, with a “head” guanylyltransferase domain, a central N7-guanine MTase, and a “bottom” 2′-O-MTase domain. This architecture illustrates the sequence of three out of the four chemical reactions involved in RNA capping described above.

Regarding (+)RNA viruses, MTase structural information at the atomic level is only available for a single genus. The flavivirus N-terminus domain (residues 1–265) of the NS5 RNA-dependent RNA polymerase harbors an RrmJ fold with an N-terminus extension able to accommodate RNA cap structures[31], [32]. This enzyme carries both N7-guanine MTase and 2′-O-MTase activities on a single domain with one shared active site[33]. Homologous domains have been crystallized for a number of flaviviruses, revealing a conserved fold and activity[34], suggesting that MTases might represent interesting targets for drug design. No other (+)RNA virus RNA cap MTase crystal structures have as yet been defined.

In 2003, the identification of the 2′-O-MTase signature sequence in the SARS-CoV genome added nsp16 to the list of putative targets for antiviral drugs[35]. Several compounds have been shown to inhibit viral MTases, such as the co-product of the MTase reaction SAH, Sinefungin, and aurintricarboxylic acid (ATA)[14], [36], [37], [38], [39]. In this paper, we report the crystal structure of the SARS-CoV 2′-O-MTase nsp16 in complex with its activator, the zinc finger protein nsp10, at 2.0 Å resolution, in conjunction with mutagenesis experiments, binding and activity assays. These results lay down the structural basis for the nsp10 function as an activator of nsp16-mediated 2′-O-MTase. We identify residues playing key roles in the nsp10/nsp16 interaction, as well as other residues involved in 2′-O-MTase catalysis and RNA binding. We also report the crystal structure of the nsp10/nsp16 complex bound to the inhibitor Sinefungin. Comparison with known cellular SAM binding sites points to the nsp16 nucleobase binding pocket as a possible target for the design of selective antiviral molecules.

## Results

### Crystallization and Structure Determination of an Active nsp16 2′-O-MTase

We observed that purified nsp16 was unstable in solution, impeding crystallogenesis. Yeast double-hybrid and co-immunoprecipitation experiments on purified SARS-CoV nsp10 and nsp16 have uncovered the reciprocal interaction of these two proteins[16], [17], [18]. Indeed, SARS-CoV nsp16 exhibits 2′-O-MTase activity only when complemented with SARS-CoV nsp10, raising the interesting possibility that nsp10 acted as a scaffold for nsp16. Co-expression of nsp10 and nsp16 using a bi-cistronic prokaryotic expression vector facilitated affinity chromatography purification and crystallization of the complex[16], [40]. Crystals diffracted to ∼1.9 Å. The position of the nsp10 protein was determined using molecular replacement with the SARS-CoV nsp10 protein structure[20] as a search model. Strong peaks in both the residual and anomalous Fourier maps confirmed the presence of two zinc ions. Nsp16 was well defined by its electron density except for two flexible loops (residues 19–35 and 135–137) with high B factors and weak or missing electron density. These loops are solvent-exposed at each side of the putative RNA-binding groove (see below). Structure determination data and refinement statistics are reported in Table 1.

**Table 1:** Crystal, collection, structure determination data and refinement statistics.

DATA	wild-type (SAH)	nsp10(Y96F)/nsp16	wild-type (SFG)
**Instrument**	SOLEIL, Proxima I	ESRF ID14-1	ESRF ID23-1
**Wavelength**	0.9792	0.9334	0.9792
Space group	C222_1_	C222_1_	C222_1_
Cell dimensions *a*, *b*, *c* (Å)	68.07 184.62 128.83	68.15 184.80 129.01	68.42 185.04 129.46
Resolution range (Å)	37.52-2.00 (2.11 – 2.00)*	45.51-2.05 (2.17 – 2.05)	45.58 -2.50 (2.64 – 2.50)
Total number of reflections	201703 (29338)	187379 (27241)	211213 (30837)
Number of unique reflections	54947 (7963)	51033 (7362)	28921 (4163)
Completeness (%)	99.7 (100)	99.6 (100)	100 (100)
*I*/σ(*I*)	7.1 (2.7)	7.5 (3.2)	12.5 (4.3)
Rsym **	0.114 (0.430)	0.112 (0.350)	0.102 (0.400)
Multiplicity	3.7 (3.7)	3.7 (3.7)	7.3 (6.7)
**Refinement (20 cycles)**			
R***	0.213	0.200	0.201
Rfree	0.227	0.234	0.215
RMSD bond length (Å)	0.007	0.007	0.011
RMSD bond angle (Å)	1.015	0.968	1.250

*Values in parentheses give the high resolution shell values.

**Rsym  = Σ |I-<I>|/Σ I.

***R = Σ||Fo|-|Fc||/Σ |Fo|.

### Structure of the nsp10/16 Heterodimer

**Figure 1:**
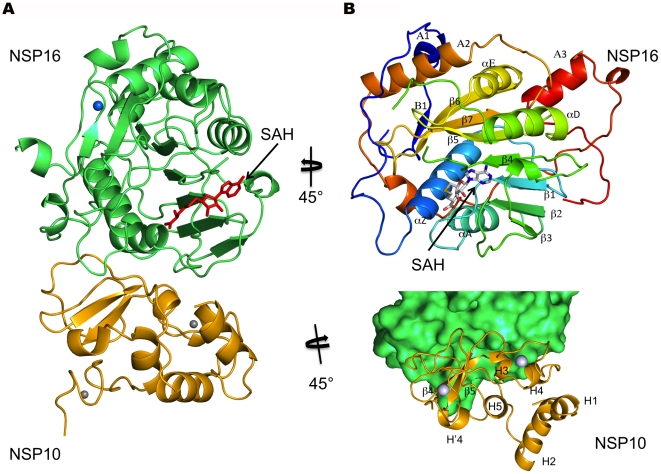
Cartoon representation of the nsp10/nsp16 complex with the reaction product SAH and metal ions. A) The nsp16 protein (green) is bound to nsp10 (yellow) through an interface which does not involve Zn ions (grey spheres) present in nsp10. One metal ion (blue sphere) is found in nsp16 on the opposite face from the active site to which a SAH molecule is found (red sticks). B) Ribbon representation of nsp16, rainbow colors from N- to C-terminus. Top: Each secondary structure element is labeled according to[25] (see also Fig. S3). The SAH molecule shown in sticks colored following atom type. Bottom: View of interface involving nsp16 (green surface) and nsp10 (yellow ribbons) showing the nsp10 secondary structure elements involved in the interface.

The heterodimer can be conveniently viewed as nsp16 sitting on top of a nsp10 monomer (Fig. 1A). The nsp10 overall structure in the complex remains essentially unchanged relative to published structures of nsp10 alone, with its N-terminus comprising two α-helices, a central β-sheet domain, and a C-terminus domain containing various loops and helices (see[20], [21], Fig. 1B). Comparison with existing crystal structures of nsp10 using DaliLite[41] rendered nsp10 atomic coordinates very similar to those of nsp10 in our nsp10/nsp16 complex. The average RMSD is about 0.77 Å in 118 residues (PDB codes 2FYG, 2G9T and 2GA6[20], [21]). This indicates that neither significant conformational change nor surface modification occurs in nsp10 when binding to nsp16. The nsp10 structural Zn^2+^ ions are not directly involved in the nsp10/nsp16 interface (Fig. 1A).

**Figure 2:**
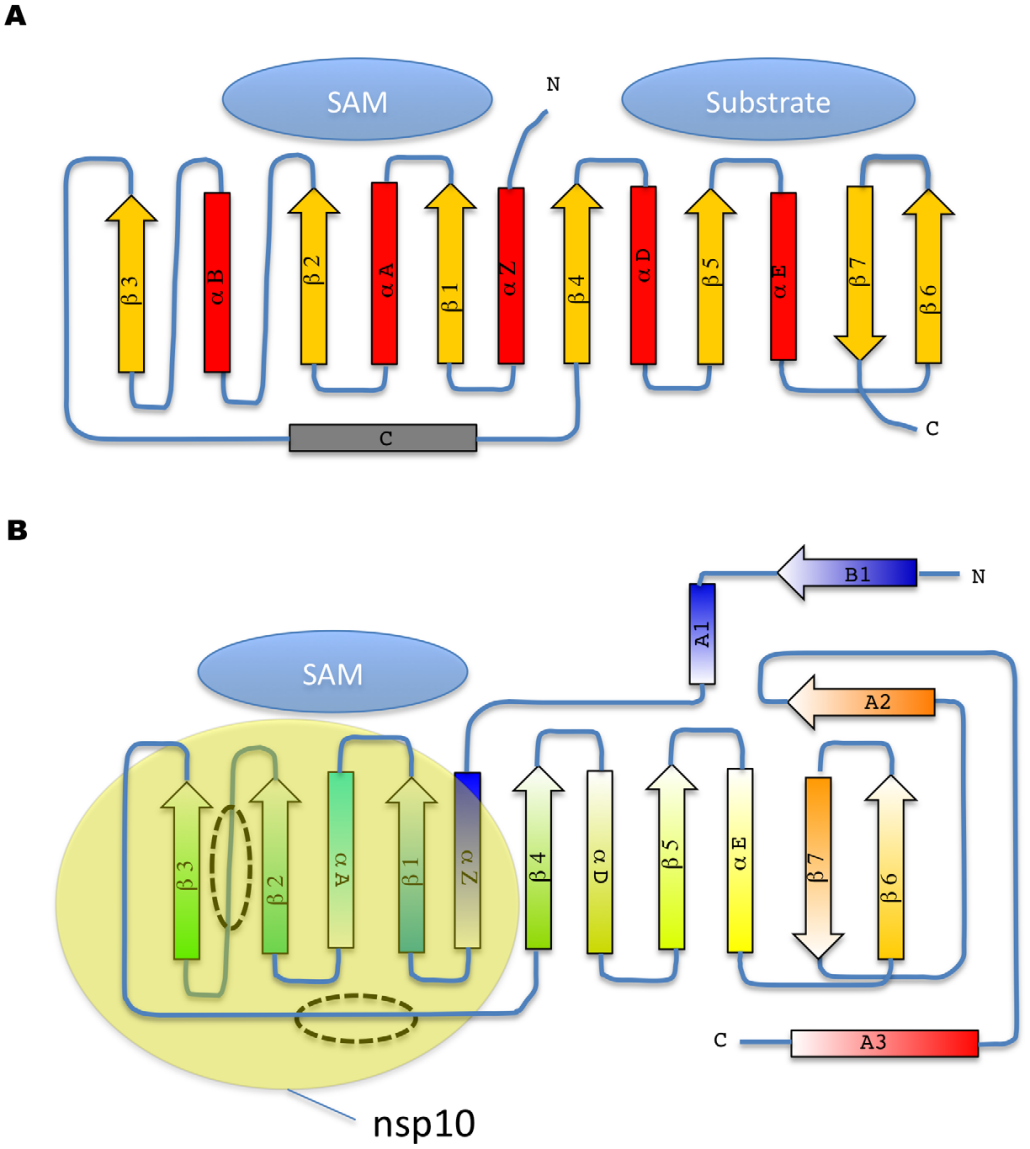
Topology diagram of MTase secondary structure elements. A) The consensus topology diagram is shown with a two domain organization for methyl acceptor substrate and SAM, as defined[25] with the catechol O-MTase and its canonical seven strand beta sheet. B) Topology diagram of nsp16 colored according to rainbow colors from N-to C-terminus as in Fig. 1B. The missing helix B and helix C are indicated by dashed ovals. The approximate general binding site of nsp10 is shown (yellow oval).

Nsp16 adopts a canonical SAM-MT fold (Figs. 1B, 2A and B), as defined initially for the catechol O-MTase[25]. The seven-stranded β-sheet MTase fold has been described as having a secondary structure topology defining two binding domains, one for SAM and the other for the methyl acceptor substrate (Fig. 2A). The nsp16 topology matches those of dengue virus NS5 N-terminal domain and of vaccinia virus VP39 MTases[27], [31]. Nsp16 lacks several elements of the canonical MTase fold, such as helices B and C (Fig. 2B).

### S-adenosylhomocysteine- and Sinefungin-Binding

**Figure 3:**
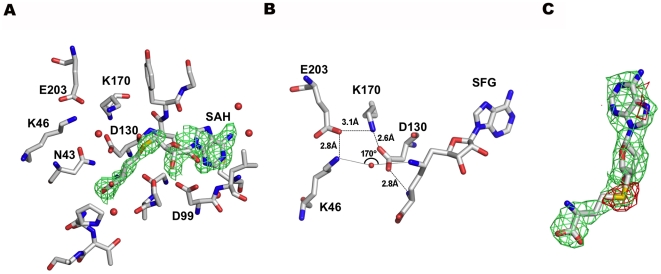
The SAM binding site of nsp16. A) SAH modeled in a simulated annealing Omit map contoured at 1σ. Carbons, oxygens, sulfur, and nitrogens are in grey, red, yellow, and blue sticks, respectively. Water molecules are shown as red spheres. B) Sinefungin bound in the SAM/SAH binding site, with main catalytic residues and the water molecule indicative of a catalytic mechanism. Colors as in A). C) SAH modeled in a 2Fo-Fc map (green) of Sinefungin contoured at 1σ with a Fo-Fc difference map (red) contoured at 3σ. A peak of negative density appears clearly on the sulfur atom of the SAH molecule showing that SAH was indeed replaced by Sinefungin. Colors as in A).

Electron density corresponding to one molecule of S-adenosylhomocysteine (SAH), the co-product of the methylation reaction, was identified in the putative SAM-binding site (Figs. 1A and 3A). Neither SAM nor SAH was added to the purification or crystallization buffers, therefore it must have been captured from the medium by nsp16 during bacterial growth. The SAH molecule is found with its adenine in an *anti* conformation and the ribose pucker in a *southern* (2′-endo/3′-exo) conformation. All the residues involved in SAM/SAH binding are absolutely conserved in coronavirus np16s (Fig. S1). Binding specificity for SAM/SAH is achieved by holding distal SAM/SAH carboxylic and amino groups through five hydrogen bonds (G81, N43, Y47, G71, and D130) (Fig. 3A and Fig. S2A). The ribose moiety is held by three hydrogen bonds involving Y132, G73, and D99. As in the case of other MTases[25], the SAH binding cleft is globally positively charged. However, an aspartic acid (D99) acts as the ribose-sensing residue with its side chain carboxyl making strong hydrogen bonds with both ribose hydroxyls (Fig. S2A). Binding of the adenine base involves few contacts. The nucleobase occupies a loose hydrophobic pocket engaging two hydrogen bonds of moderate strength with side chain and main chain atoms of conserved residues D114 and C115, respectively.

Soaking the crystals into a Sinefungin-containing buffer captured this MTase inhibitor in the SAH binding site almost perfectly superimposable on SAH (Fig. 3B and Fig. S2B). Binding involved the same residues and contacts as SAH. Inhibition of the MTase reaction by Sinefungin therefore probably occurs competitively. The Sinefungin amino group quasi-isosteric to the donated SAM methyl group indicates a cavity where the 2′-hydroxyl of the capped RNA is expected to bind. Lining this empty substrate cavity are the residues proposed to be involved in the catalytic reaction: K46, D130, K170, and E203[16]. Alanine substitutions in the catalytic tetrad (K46, D130, K170, or E203) almost completely block 2′-O-MTase activity without jeopardizing binding to nsp10 (Table 2, and [16]). Several SAM-binding residues (N43, G73, D99 and Y132, Fig. S2A) were substituted by alanine. Although they conserve their specific nsp10 binding properties, indicating that they are correctly folded, they all show a drastically reduced MTase activity (Table 2), validating the structural description of the nsp10/nsp16/SAH ternary complex.

**Table 2:** Mutational analysis, complex formation, and enzyme activity of the nsp10/nsp16 complex.

Number	Function*	Mutant	% associated**	% MTase act. ***
1 = Wild-Type	Wild-TypE	Wild-Type	100	100
2	Catalytic	K46A	97	1
3	Catalytic	D130A	104	2
4	Catalytic	K170A	95	0
5	Catalytic	E203A	93	0
6	SAM-BS	N43A	144	11
7	SAM-BS	G73A	119	18
8	SAM-BS	D99A	57	0
9	Mg^2+^-BS	T58A	123	57
10	Mg^2+^-BS	T58N	110	30
11	Mg^2+^-BS	T58E	54	1
12	Mg^2+^-BS	S188A	102	28
13	RNA/SAM-BS	Y132A	86	5
14	RNA/SAM-BS	Y132T	95	5
15	RNA/SAM-BS	Y132F	123	9
16	RNA/SAM-BS	Y132H	106	0
17	RNA-BS	Y30A	89	1
18	RNA-BS	Y30F	113	6

Interface mutants				
Number	Location*	Mutant	% associated**	% MTase act. ***
19	Patch I	I40A	84	8
20	Patch I	M41A	122	4
21	Patch I	V44A	113	1
22	Patch I	T48A	123	20
23	Patch II	V78A	3	0
24	Patch II	R86A	61	0
25	Patch II	Q87A	114	62
26	Patch III	V104G	6	4
27	Patch III	D106A	101	38
28	Patch IV	L244A	30	0
29	Patch IV	M247A	4	0

*as inferred from the crystal structure.

**nsp10/nsp16 complex formation as determined using pull-down experiments, SDS-PAGE analysis, and quantitation (see Methods).

***The % of MTase activity was determined using filter binding assays (see Methods) relative to wild-type.

SAM-BS: S-adenosylmethionine binding site.

Mg^2+-^BS: Magnesium binding site.

RNA-BS: RNA binding site.

### A Mg^2+^ Cation is Present in nsp16, Outside the Active Site

**Figure 4:**
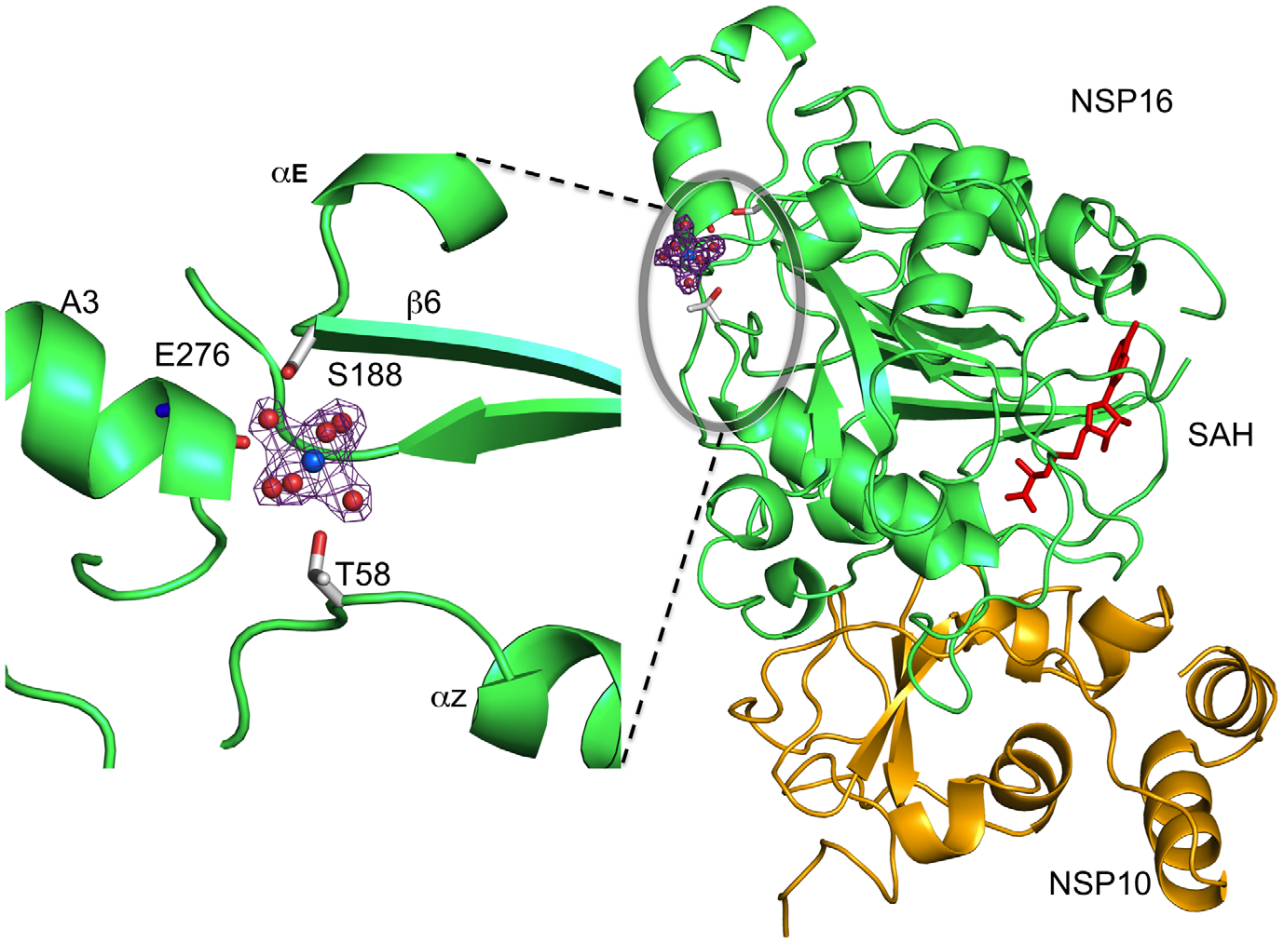
Position and coordination of a metal ion. Left, the putative Mg^2+^ ion (blue sphere) is shown solvated in its first atomic shell by six water molecules (red spheres). The corresponding 2Fo-Fc electron density map (contoured 1σ) with a cross shape is shown in purple. Residues of Nsp16 involved in coordination of Mg^2+^ ion via water molecules are labeled. Right, a global view of the bound metal ion in its electron density on the opposite side from the SAH molecule (red sticks). Nsp16 is shown as green ribbons, nsp10 as yellow ribbons with its two bound Zn^2+^ as grey spheres as in Fig. 1.

We recently reported[16] that nsp10/nsp16 MTase activity requires Mg^2+^. Although the crystallization buffer contains Mg^2+^, we were unable to locate any such cation in the nsp16 active site. In enzyme activity assays, the Mg^2+^ ion can be substituted by Mn^2+^ or Ca^2+,^ but not Zn^2+^ (data not shown, see also[16]). A peak of electron density presumably corresponding to Mg^2+^ is localized onto nsp16, distant from the SAH-binding cavity. The Mg^2+^ coordination mode is through six first-shell water molecules in an octahedral geometry (Fig. 4). Binding *via* water molecules, involves T58 and S188 side chain hydroxyls and the main chain carbonyl of E276. Since there are no carboxylic acids involved in binding this cation, it was suspected that its presence resulted from the crystallization procedure[42], with no biological relevance. However, the T58A, T58N, T58E and S188A substitutions show 43, 70, 99 and 72% loss of activity, respectively (Table 2), with no significant effect on the stability of the nsp10/nsp16 complex except for T58E whose association was 54 % that of wild-type. These residues are located on three distinct structural elements at the C-terminus of helix Z (T58), the N-terminus of β6 (S188), and in the central part of helix A3 (E276), respectively (Fig. 4 and Fig. S3). The cation may thus hold these elements together.

### The Putative RNA Binding Site: Mutagenesis and Effect on Activity

**Figure 5:**
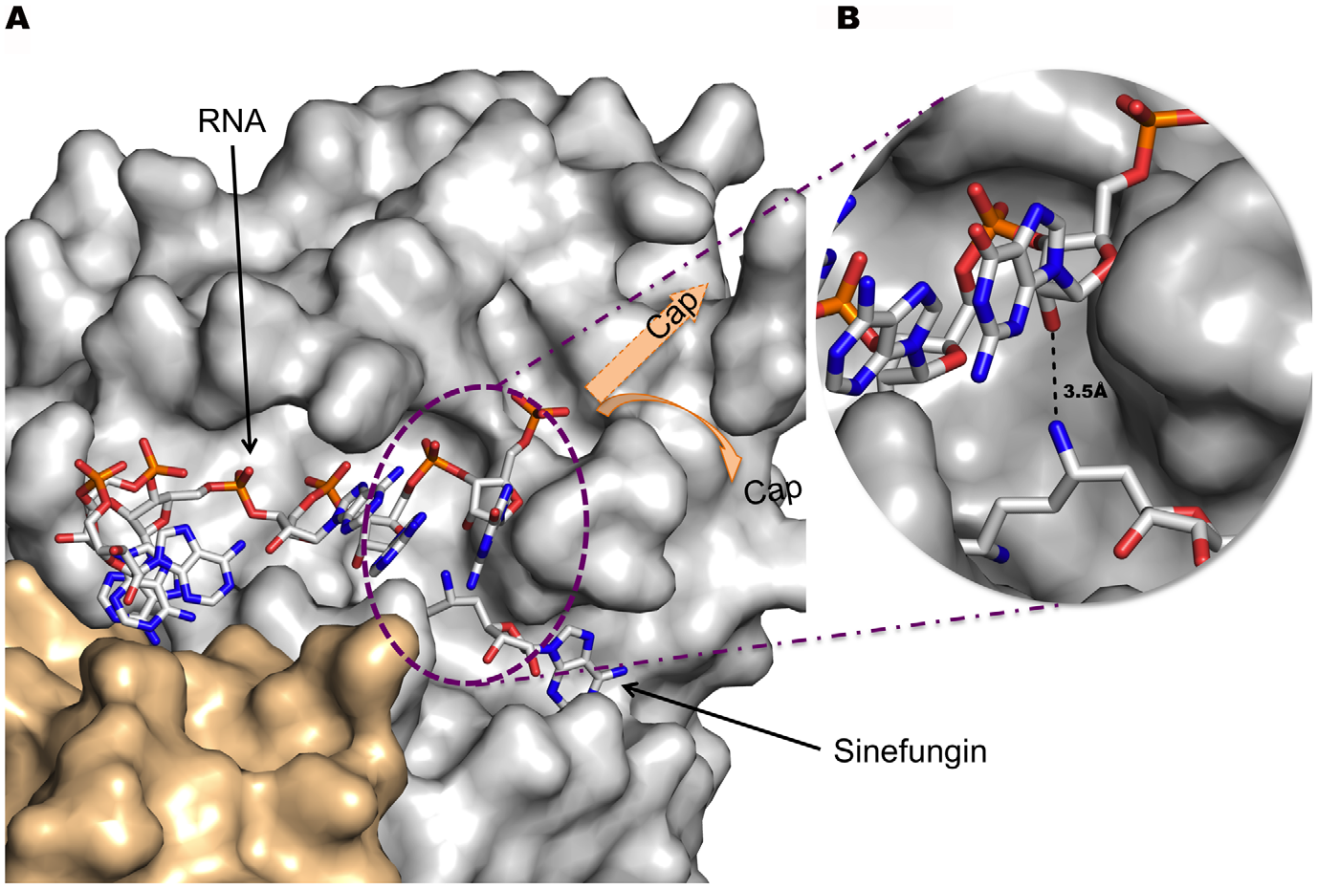
Stick model of RNA bound to the nsp16 RNA binding groove and Sinefungin in the methyltransferase active site. A) In this representation, Sinefungin was preferred over SAH because one of its NH_2_ groups approximates the direction of a transferred CH_3_ group from the SAM substrate. Carbon is white, oxygen is red, nitrogen is blue and phosphorous is orange. Nsp16 and nsp10 are rendered as a solvent-accessible surface colored grey and wheat respectively. The Sinefungin molecule defines the methyltransferase active site. Missing residues in the 135-137 loop (see text) are indicated by a shaded blue dotted box. Position of Y30 and Y132 are indicated. Y30 generated poor electron density (see « Methods ») and its aromatic ring position has been manually adjusted before generation of this image. B) Close caption of the methyltransferase active site showing distance between the NH2 of Sinefungin to the 2′-O of the ribose of the first base, thus mimicking the position of the methyl of the S-adenosylmethionine.

The nsp10/nsp16 complex absolutely requires an N7-methyl guanine capped RNA substrate to exhibit MTase activity[16]. The structural basis for the preferential binding to methylated N7-guanine versus non-methylated caps has been elucidated in four cases, those of VP39[27], eIF4E[43], CBC[44], and PB2[45] proteins (PDB codes 1AV6, 1EJ1, 1H2T, and 2VQZ, respectively) bound to cap analogues or capped RNAs. In these cases, the methylated base specificity is achieved through increased binding energy resulting from the stacking of the N7-methyl guanine between parallel aromatic residues of the cap binding protein. The presence of the methyl group greatly enhances π-π stacking, providing a dominant effect over unmethylated guanine[46]. Despite numerous attempts, cap analogues (m7GpppA, GpppA, m7GpppG, GpppG) and short capped RNA substrates (m7GpppA(C)_n_) could neither be co-crystallized with nsp10/nsp16 nor soaked and bound onto preformed nsp10/nsp16 crystals. However, the atomic coordinates of the N7-methyl guanine RNA oligomer in complex with VP39[47] provided data from which a model of RNA binding to the nsp16 protein was derived. SAM molecules identified in both structures were superimposed, and the VP39-bound RNA was positioned onto the nsp16 structure. After minimal manual adjustments not exceeding 5 Å, the VP39 RNA was a reasonably good fit into an nsp16 hydrophobic groove radiating from the catalytic site (Fig. 5), establishing very few contacts with nsp10. We note that the protein side diametrically opposite to the proposed hydrophobic RNA binding groove is highly positively charged (not shown), an observation that may account for the difficulty of achieving experimental RNA binding in the proposed RNA binding site. In the absence of robust data to guide docking of the guanine cap, the m7Gpp cap structure was not positioned in the structure but two possible N7-methylated cap guanine binding areas are indicated by arrows (Fig. 5). The first transcribed nucleotide together with its ribose receiving the methyl group fit well in the active site (Fig. 5, panel B) as predicted in the proposed mechanism. The same holds for the immediately preceding three nucleotides. The base of the first transcribed nucleotide may be held by contact with P134 and Y132, bending the extending RNA cap structure. Accordingly, the substitution of Y132 greatly depresses MTase activity (Table 2). We also note that Y132 is located in the vicinity of a highly mobile loop (residues 135-138) not always visible in our crystal structures suggesting that this loop may move in order to wrap the triphosphate moiety of the RNA cap and/or the RNA cap itself. The solvent exposed side chain of Y30 may also participate in RNA binding. In the model, the highly mobile side chain of Y30 was flipped out in an alternative conformation in order to open the groove. In that position, Y30 should specifically contact the third transcribed nucleotide. Our mutagenesis data confirms the importance of Y30 since its replacement with either Ala or Phe severely impairs MTase activity without affecting the interaction with nsp10 (Table 2).

### Interface of the Heterodimer

**Figure 6:**
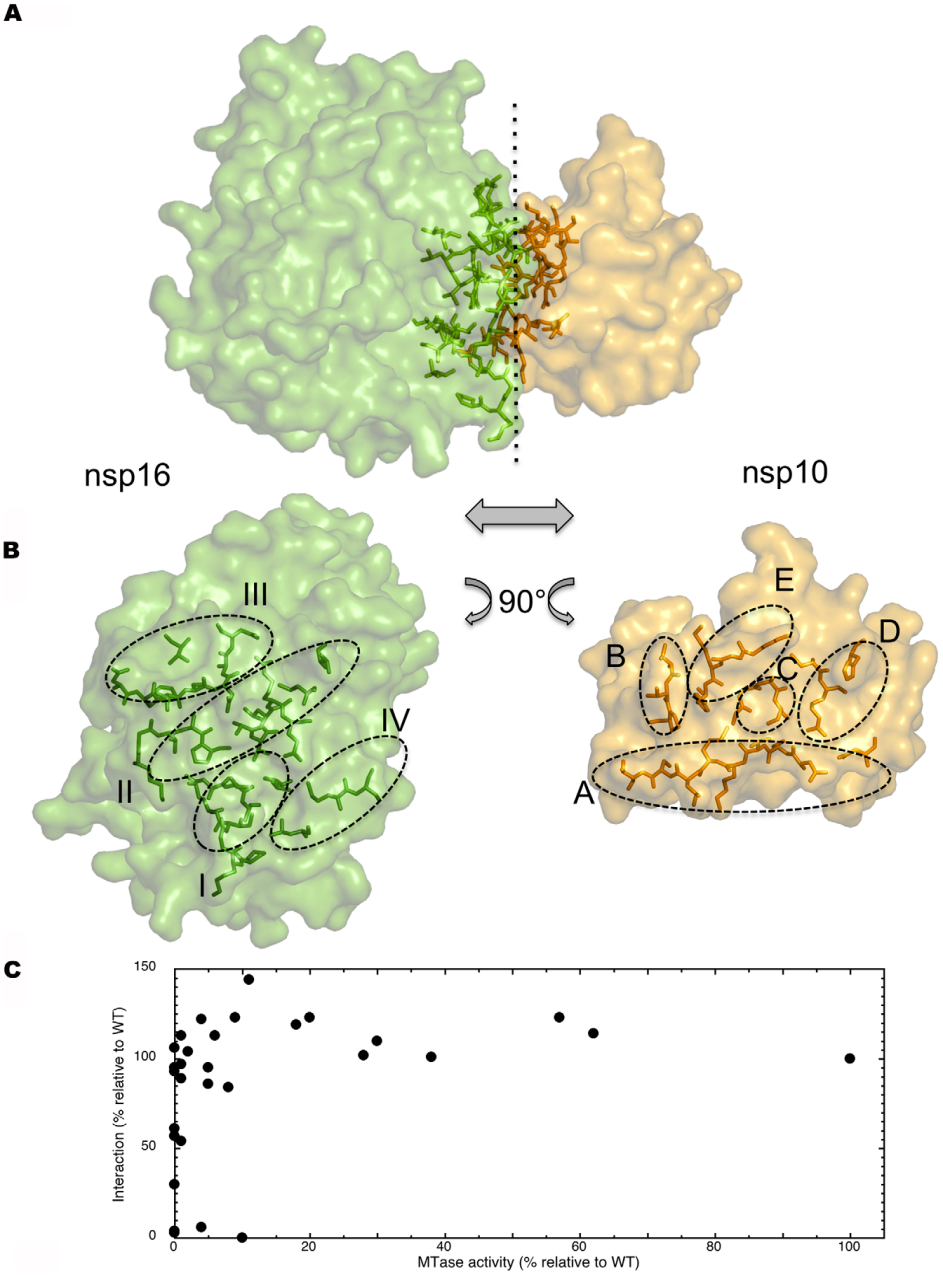
Detailed definition of nsp16 and nsp10 elements involved in the interface. A) nsp16 (left) and nsp10 (right) are rendered as a solvent-accessible surface colored green and yellow, respectively. Residues involved in the interface are rendered as sticks with the same color code, in transparency into respective proteins. B) Separate representation of the nsp16 (left) and nsp10 (right) interface. Nsp16 residues defining patch I, II, III, and IV are rendered as sticks as above in transparency into nsp16. Nsp10 residues defining patch A, B, C, D, and E are rendered as sticks as above in transparency into nsp10. C) A plot of MTase activity as a function of nsp10/nsp16 complex formation using the data reported in Table 2.

All nsp10 secondary structure elements but helices 2 and 5 contact nsp16 (Fig. 1). The nsp10 contact points can be viewed as 5 small patches A to E (residues 40–47, 57–59, 69–72, 77–80, and 93–96, respectively, Fig. 6A). In turn, these five patches contact most of the nsp16 SAM-binding structural elements in 4 areas, I to IV (Fig. 6B, Fig. S3)), mainly involving β2, β3, αA, αZ, and for area IV, the loop connecting helices A2 and A3 at the C-terminus (Figs. 1 and 6). In total, the interface of the heterodimer involves 53 residues, 23 and 30 from nsp10 and nsp16, respectively. In nsp10, a single residue (Asn10, at the edge of the interaction surface) is not conserved out of 23 (4.3 %), whereas in nsp16 there are 8 non-conserved residues out of 30 (26.7 %) (Fig. S1). The interface has a buried surface area of 1820 Å^2^, with nsp10 contributing to 930 Å^2^ and nsp16 to 890 Å^2^.

Four nsp10 patches included in the 5 interaction patches identified here were recently mapped using reverse yeast two-hybrid methods coupled to bioluminescence resonance energy transfer and *in vitro* pull-down assays (see[18] and below). To probe the observed crystal structure of the interface further, we engineered 5 new nsp10 alanine mutants (N40A, L45A, T58A, G69A, and H80A, see Table S1) sitting in patches A, B, C and D. Whereas T58A, G69A and H80A showed limited effect on nsp16 binding, N40A reduced it to 64% of wild type affinity, and L45A almost abrogated it. The crystal structure indicates that the nsp10 interface proposed by Lugari *et al.* [18], is a correct and conservative estimation, as the interface also includes L45 belonging to patch A. We also confirm the positive co-relation of the detected nsp10/nsp16 interaction with MTase activity. In no instance can nsp16 be active in the absence of nsp10/16 complex formation.

The Y96 position is of particular interest. Alanine substitution (Y96A) abrogates interaction whereas a phenylalanine (Y96F) increases both interaction and MTase activity[18]. In order to understand how residue 96 plays such a pivotal role, we determined at 2.0 Å resolution the crystal structure of this nsp10(Y96F)/nsp16 complex (Table 1). Strikingly, the absence of the hydroxyl group does not alter the topology of the interface. Wild-type and Y96F residues superimpose without significant difference at all atomic positions (not shown). Either Y96 or F96 is in direct contact with nsp16 helix αZ, which carries the catalytic residue K46. Detailed surface analysis using PISA indicates that the position of K46 in Y96F nsp10 is identical to that of K46 in wild-type nsp10, ruling out a better alignment of catalytic residues of the Y96F mutant. The nsp10(Y96F)/nsp16 differs from wild-type nsp10/nsp16 in the SAH binding site, though. When compared to that of wild-type, the SAH occupancy is much lower (∼0.3 versus ∼1), leading to poor density definition. SAH is known to be a fairly good inhibitor of the methylation reaction. Therefore, a lower binding affinity might translate into less end-product inhibition, and account for the observed increased activity. We measured the affinity of nsp16 for SAH using fluorescence spectroscopy, but no significant differences were found (not shown). Likewise, the MTase inhibition pattern by SAH was identical for wild-type and nsp10(Y96F)/nsp16 (not shown). We therefore infer that the previously observed ∼10-fold increased stability of the heterodimer[18] may be responsible for the increased activity. A more hydrophobic character of the interaction may appear upon the loss of the tyrosine hydroxyl which, in the wild-type protein, was not engaged in any polar contact. We thus attribute the increased activity of the nsp10(Y96F)/nsp16 complex relative to wild-type to a stronger equilibrium association of nsp10(Y96F) with nsp16 than that of wild-type nsp10 with nsp16.

Mutation analysis was also conducted on nsp16 residues presumably involved in the interface and interfacial activation (Tables 2 and S2). Several mutants (V78A, V104A, L244A, M247A) in patches II, III and IV completely disrupt the nsp10/nsp16 complex and annihilate nsp16 MTase activity. Interestingly, we also identified nsp16 mutants still interacting with nsp10, but with a strongly reduced 2′-O-MTase activity (I40A, M41A, V44A, T48A, Q87A, D106A) suggesting, that these mutations in the nsp10/nsp16 interface may alter the fine positioning of catalytic residues without any significant effect on nsp10 binding. Accordingly, most of these mutants are localized in αZ helix of patch I which contains the K46 catalytic residue. On the other hand, patch II and III mutants tend to have more mitigated phenotypes, yielding to full-blown interaction with only about half of the expected activity. Finally, patch IV mutants were totally inactive. Using all mutants reported in Table 2, a plot (Fig. 6C) of interaction versus activity shows that the nsp10/nsp16 interaction is strictly required to obtain significant nsp16 MTase activity.

## Discussion

### Mechanism of 2′-O-Methyltransfer

The SARS-CoV RNA cap 2′-O-MTase is a heterodimer comprising SARS-CoV nsp10 and nsp16. When bound to nsp10, nsp16 is active as a type-0 RNA cap-dependent 2′-O-MTase, ie., active only when the cap guanine is methylated at its N7 position[16]. The nsp10/nsp16 crystal structure shows that nsp16 adopts a typical fold of the S-adenosylmethionine-dependent methyltransferase family as defined initially for the catechol O-MTase[25]. A good alignment (170°) is found between the SAH sulfur atom, a water molecule present in both SAH- and sinefungin-bound nsp16 structures, and the K46 ε-amino group (Fig. 3A and B). This geometry provides interesting hints for a catalytic mechanism, as the positions of the catalytic residues (K46, D130, K170, E203) match spatially those of the vaccinia virus VP39 2′-O-MTase[47]. At the initial stage of the reaction the 2′-hydroxyl of the capped RNA substrate would occupy the position of the water molecule. In turn, E203 and K170 decrease the pKa of the K46 ε-amino group that becomes a deprotonated general base (-NH_2_) able to activate the RNA 2′-hydroxyl at neutral pH. In VP39, K175 has been identified as the general base catalyst[47] with a pKa depressed by ∼ 2 pH units by the neighbouring D138 and R209 residues[48]. These findings indeed suggest a related mechanism: once K46 has activated the 2′-hydroxyl group, the 2′-oxygen would produce an *in line* attack through a SN2-like mechanism onto the electrophilic SAM methyl group. The methyl group would pass through a pentavalent intermediate with the 2′-O and sulfur at apical positions. D130 is positioned to stabilize the transient positive charge on the donated methyl atom of SAM before the sulfur recovers a neutral electric charge during SAH generation (Fig. 3B).

### Role of Mg^2+^ on MTase Activity

Unlike most SAM-dependent MTases, the SARS-CoV nsp10/nsp16 enzyme requires a divalent cation, either magnesium, manganese or calcium[25]. We have found that this cation does not reside in the active site. Instead, the cation is coordinated through water molecules by three residues located on three distinct structural elements. It is thus possible that one divalent cation, presumably Mg^2+^, present in the host cell at millimolar levels, plays a structural role in holding these three nsp16 structural elements together and so regulate the enzyme activity. It is intriguing that T58A is more active than T58N or T58E that can still bind the water that chelates to the metal. Alternatively, it is possible that divalent cations such as Mg^2+^ or Ca^2+^ act as a phosphodiester charge shield to allow RNA binding in the hydrophobic binding groove[49].

### MTase Activity is Regulated via Protein-Protein Interactions

The main regulation mechanism of nsp16 is through its physical association with nsp10. Nsp16 is unstable in solution, and nsp10 acts as a scaffold for nsp16, yielding a stable dimer active as an RNA cap-dependent (nucleoside-2′-O)-MTase. The complex is assembled through a ∼890 Å^2^ contact surface in nsp16, an area typically in the intermediate zone differentiating strongly from weakly associated dimers[50]. This finding is consistent with a Kd estimated at ∼0.8 µM[18] that qualifies the nsp10/nsp16 complex as a rather weak heterodimer. The nsp10 interaction surface identified in the crystal structure was confirmed by site-directed mutagenesis and overlaps that previously identified by indirect methods[18]. Remarkably, the nsp10 surface in the nsp10/nsp16 complex is essentially identical to that of uncomplexed nsp10 crystallized alone by others[20], [21](Fig. S4). It is therefore reasonable to see this heterodimer as a non-permanent species which would tolerate nsp10 or nsp16 engaging in interactions with other partners. This notion is actually in line with the involvement of nsp10 in a network of protein-protein interactions that we and others have proposed[17], [19]. Donaldson *et al.* [23] have engineered mutations in nsp10 using reverse genetics. Out of eight mutations that turned out to be in the nsp10/nsp16 interface (this work), five, two and one rendered lethal, debilitated, and viable phenotypes, respectively[23]. Interestingly, the nsp10(Q65E) mutant providing a temperature-sensitive phenotype[22], [23] does not map in the nsp10/nsp16 interface, confirming that nsp10 has a pleïotropic role.

Our mutagenesis analysis shows that the formation of an nsp10/nsp16 complex is a pre-requisite for MTase activity (Fig. 6C) indicating that physical association of nsp10 and nsp16 is essential to activate nsp16 2′-O-MTase activity and foster efficient virus replication. We note that most interface mutants exhibit a severe loss of their 2′-O-MTase activity, whereas the apparent association affinity is often only modestly affected. That minor changes in the interface translate into potent effects is also dramatically illustrated by the Y96F mutation, where the loss of a single hydroxyl provokes a significant change in affinity[18]. Remarkably, it is not the most active complex that was selected in nature, since the nsp10(Y96F)/nsp16 complex is both more stable and more active than the wild-type heterodimer (this work and[18]). This is yet another observation hinting at the involvement of nsp10 in protein-protein interaction networks including other partners than nsp16, such as nsp5 and nsp14[17], [19]. In most other coronaviruses, the nsp10 residue at position 96 is a phenylalanine. It would be interesting to determine whether this polymorphism is relevant to the SARS-CoV pathogenicity at any (direct or indirect) level, or if compensating polymorphisms in other coronaviral nsp10 (or nsp16) restore a weaker nsp10/nsp16 association equivalent to that of the SARS-CoV pair. Since a *bona fide* viral RNA cap is key in evading the host cell innate immunity[4], [51], a minimal level of 2′-O-MTase activity would be expected to be critical to virus survival.

MTase activation through dimerisation of two viral protein partners has already been reported in the case of the vaccinia virus D1/D12 N7-guanine MTase[28]. However, the activating D12 subunit does not contact the D1 subunit through a homologous surface mainly defined by canonical αA and αZ helices. Rather, the D1/D12 activation surface would be located at a 90° clockwise rotation relative to the nsp10/nsp16 interface depicted in Fig. 1A. In the case of dengue virus, the bi-functional N7-guanine and 2′-O-MTase is part of the N-terminus of the dengue NS5 protein. Based on reverse genetic data and modeling[52], the MTase domain would be associated with the Pol domain through an interface topologically similar to that of nsp10/nsp16, *i.e.*, involving mainly helices αA, αZ and strands β2 and β3 as depicted in Fig. 1A.

We have previously shown that the nsp10/nsp16 is only active as N7-guanine methylated capped RNA, implying that RNA cap methylation obeys to an ordered sequence of events where nsp14-mediated N7-guanine methylation precedes nsp10/nsp16 RNA 2′-O methylation[16]. In the absence of data regarding the RNA substrate, we built a model of RNA binding based on that of the vaccinia virus VP39 ternary complex structure. Interestingly, our model proposes that the RNA interacts only with nsp16 residues, in keeping with what was recently suggested based on RNA binding assays[14]. Although the position of the cap structure on the nsp16 surface remains to be determined, our model suggests a well-defined position for the ribose of the first transcribed nucleotide in the active site. In agreement with mutagenesis analysis, the model also suggests that the transcribed RNA 5′-end stacks between Y132 and Y30. Furthermore, this model is consistent with the observation that coronavirus MTase requires RNA substrates of at least 3 transcribed nucleotides in length[14]. It is also worth to know that a comparison of nsp16 and VP39 electrostatic surfaces reveals that the putative RNA-binding groove of nsp16 is mostly hydrophobic, whereas the VP39 RNA-binding groove is positively charged. This variation would imply a change in the nature of the RNA/protein interaction.

### Binding of Sinefungin to nsp16/nsp10 Suggests Feasibility of a Drug Design Approach to Inhibit MTase Activity

Viral MTases are increasingly evaluated as potential drug design targets[34], [37], [53]. We have crystallized the inhibitor Sinefungin with the nsp10/nsp16 complex. Sinefungin exhibits an IC_50_ of 0.74 µM, 16-fold lower than that of SAH as reported by Bouvet *et al.* [16] using purified nsp10/nsp16. Analysis of the structure suggests a likely mechanism of action that also accounts for the observed inhibitory effect of this drug. We note that the adenine nucleobase does not fit snugly into its binding pocket, raising interest regarding structure-based drug design. Preliminary examination of eukaryotic non-viral MTase structures from main classes as defined in Martin and McMillan[25] indicates that the SAH adenine is bound tighter in any of the latter enzymes than in the nsp16 SAM-binding site, indicating a possible breach to achieve anti-coronavirus selectivity with a small molecule inhibitor of nsp16.

In conclusion, the crystal structures presented here extend our general understanding of the mechanism and regulation of viral RNA cap MTases in (+)RNA viruses, and point to both the nsp10/nsp16 interface and the substrate binding sites as putative antiviral targets.

## Methods

### Crystallization and Data Collection

Both nsp10 and nsp16 were expressed from the same dual expression vector pmCOX [16]. Nsp10 had a N-terminal strep-tag (WSHPQFEK), and nsp16 a N-terminal hexa-histidine tag. The purification and crystallogenesis of the nsp10/nsp16 complex was performed as described in [40]. Typical crystals of the wild-type nsp10/nsp16 appear in hanging drops after 24 h at 20°C in 0.1 M CHES pH 9, 1.52 M MgCl_2_ hexahydrate. Crystals (a = 68.53 Å, b = 184.74 Å, c = 129.01 Å, C222_1_) contain one nsp10/nsp16 complex per asymmetric unit, with a solvent content of 70 % and V_m_ of 4.17 Å^3^/Da. Crystals of nsp10(Y96F)/nsp16 were grown in 67 mM CHES pH 8.5, 0.99 M MgCl_2_ hexahydrate, 33 mM Tris-HCl, 8.3 % PEG 8000. Both crystallization conditions yielded crystals diffracting to 1.9 Å when exposed to synchroton radiation at the ID14-1 beamline of the European Synchrotron Radiation Facility, Grenoble, France. Crystals were cryo-cooled in the same buffer supplemented with 15 % glycerol. Crystal soaking was performed in the same buffer supplemented with 5 mM SAH or Sinefungin during 24 h.

### Structure Determination and Refinement

The position of the nsp10 protein was unambiguously determined by molecular replacement using the program PHASER[54] with the nsp10 protein (2FYG), as search probe[20]. Strong peaks in both the residual and anomalous Fourier maps confirmed the presence of two Zinc ions at the expected positions within the nsp10 protein, thus giving confidence in the validity of the MR solution. Phases calculated from this partial model were combined with SAD phases from the Zn atoms using PHASER. To ameliorate the resulting low quality density map, phases were improved with PARROT[55]. An initial model, comprising both nsp10 and nsp16, was automatic built by successive use of BUCCANEER[56] and ARP/wARP[57]. The resulting model was subject to several cycles of manual rebuilding using COOT [58] and refinement with REFMAC [59]. The protein structure model could be built, except the strep and hexahistidine tags. In nsp16, density was too weak for the mobile, solvent exposed nsp16 loop 136–139, Y30 (see “Results”), and 2 and 6 residues in N- and C-terminus, respectively. Likewise, nsp10 solvent exposed 9 and 8 residues in N- and C-terminus were missing, respectively.

Overall, the chain traces are unambiguous, with clear electron density including for a single SAH residue bound to the nsp16 protein. Solvent accessible surfaces were calculated using program AREAIMOL [60] with a 1.7 Å radius sphere as the probe (Table 1) and values rounded to the nearest 5 Å^2^. Conformational differences were analyzed using the DynDom server (http://www.cmp.uea.ac.uk/dyndom/main.jsp). Figures were created using PYMOL (http://www.pymol.org). The coordinates of the wild-type/SAH, mutant, and wild-type/Sinefungin structures have been deposited at the Protein Data bank under PDB codes 2XYQ, 2XYV, and 2XYR, respectively.

### RNA Cap Structure Modeling

The modeling of the RNA cap structure in the nsp10/nsp16 complex structure is derived from the analysis of the structure of the vaccinia virus methyltransferase VP39 crystallized in complex with a capped RNA and a S-Adenosylhomocysteine[47] (SAH) (pdb code: 1AV6). The two structures are manually aligned using COOT[58] based on the position of SAH binding sites, as well as SAH, and Sinefungin (SFG) molecules. The RNA binding site of VP39 is only partly overlapping that of nsp16 whilst the shape of the cavity is similar; thus local adjustments necessary to accommodate the RNA molecule in its binding groove were done manually using COOT. The side chain of tyrosine 30 of nsp16initially pointed to the putative RNA binding site, preventing any *bona fide* modeling. In order to fit the RNA molecule in the cavity, an alternative conformation was sought for this side chain. The second most common conformation for the tyrosine side chain was selected. Due to the biochemistry data and surface electrostatic analysis, it is not possible to describe with certainty the final position of the cap, thus the cap was removed and replaced by arrows symbolizing possible positions. No other modification was performed on the RNA, the Sinefungin molecule or the nsp16 structure.

### Plasmids

The SARS-CoV nsp10 and nsp16-coding sequences were amplified by RT-PCR from the genome of SARS-CoV Frankfurt-1 (accession number AY291315) as previously described[16]. The nsp10 and nsp16 genes (encoding residues 4231–4369, 5903–6429, and 6776–7073 of replicase pp1ab) were cloned into a Gateway modified dual-promotor expression plasmid and in the gateway pDest 14 expression vector. In this backbone, SARS CoV nsp10 can be expressed under a tet promoter and encodes a protein in fusion with a N-terminal strep tag, whereas nsp16 is expressed under a T7 promoter and encodes a protein in fusion with a N-terminal hexahistidine tag. The mutants were generated by PCR using the Quickchange site–directed mutagenesis kit (Stratagene), according to the manufacturer's instructions.

### Reagents

AdoMet and cap analogs GpppA and ^7Me^GpppA were purchased from New England BioLabs, the[^3^H]-AdoMet was purchased from Perkin Elmer and Sinefungin (adenosylornithine) from Sigma-Aldrich.

### Expression and Purification of SARS-CoV nsp10, nsp16 and nsp10/nsp16 Complex

*E. coli* C41 (DE3) cells (Avidis SA, France), containing the pLysS plasmid (Novagen), were transformed with nsp10 or nsp16 cloned in pDest14, or nsp10/nsp16 cloned in pmCox, and grown in 2YT medium supplemented with appropriate antibiotics. The expression of strep-tagged nsp10 or 6His-tagged nsp16 mutants was induced (DO_600_ = 0.6) by adding 50 µM IPTG, and the expression of the nsp10/nsp16 complex by adding 50 µM IPTG and 200 µg/L of anhydrotetracycline. After an incubation for a 16 h at 24°C, the cell were pellets, frozen and resuspended in lysis buffer (50 mM HEPES, pH 7.5, 300 mM NaCl, 5 mM MgSO_4_, 5 mM β-mercaptoethanol (only for nsp10) supplemented with 1 mM PMSF, 40 mM imidazole, 10 µg/ml DNase I, and 0.5% Triton X-100. After sonication and clarification, proteins were purified either by IMAC (HisPurTM Cobalt Resin; Thermo Scientific) chromatography[16] (nsp10 mutants and nsp16 mutants), and the nsp10/nsp16 complex was purified by using Strep-Tactin sepharose (IBA Biotagnology) as previously described[16]. All purified proteins were analyzed by SDS-PAGE. The binding of wild-type nsp10 to mutant nsp16, and that of mutant nsp10 to wild-type nsp16 was quantified using ImageJ as described[16].

### Radioactive Methyltransferase Assay

MTase activity assays were performed in 40 mM Tris-HCl, pH 8.0, 5 mM DTT, 1 mM MgCl_2_, 2 µM ^7Me^GpppAC_5_ or GpppAC_5_, 10 µM AdoMet, and 0.03 µCi/µl [^3^H]AdoMet (GE Healthcare). Short capped RNAs (^7Me^GpppAC_5_, GpppAC_5_, were synthesized *in vitro* using bacteriophage T7 DNA primase and were purified by high-performance liquid chromatography (HPLC) as previously described[61]. In the standard assay, nsp10 and nsp16 were added at final concentrations of 600 nM, and 200 nM, respectively, and the amount of ^3^H-CH_3_ transferred onto ^7Me^GpppAC_5_ substrates was determined by filter binding assay as previously described[16].

## Supporting Information

Figure S1 Sequence alignment and amino acid conservation in coronavirus nsp16. The alignment of coronavirus nsp16 sequences was generated with Muscle program (http://www.ebi.ac.uk/Tools/msa/muscle/), and the resulting alignment converted using the ESPript program, (http://espript.ibcp.fr/ESPript/cgi-bin/ESPript.cgi). Residues that are conserved in all or >70% sequences are boxed in red and yellow, respectively. National Center for Biotechnology Information (NCBI) accession numbers for replicase polyprotein sequences that include nsp16 are as follows: Severe acute respiratory syndrome virus SARS-CoV Frankfurt isolate (SARF), Severe acute respiratory syndrome virus SARS-CoV Tor2 isolate (SART), NP_828873.2; Turkey coronavirus (Turk), YP_001941189; Infectious Bronchitis Virus (IBV), NP_066134; Feline Coronavirus (Feli), YP_239426;Porcine Transmissible Gastroenteritis Coronavirus (PTGC), P18457; Transmissible Gastroenteritis coronavirus (TGEV), NP_840013; Porcine epidemic diarrhea virus CV777 (PEDV), NP_839969; Bat coronavirus 512/2005 (BATC), YP_001351683; Human coronavirus NL63 (NL63), AAS58176; Human coronavirus 229E (229E), NP_073549; Murine hepatitis virus strain JHM (MurJ), YP_209243; Mouse Hepatitis virus strain A59 (MA59), NP_740613; Human coronavirus HKU1 genotype A (HKU1), YP_460023; Human enteric coronavirus 4408 (4408), ACJ35483; Bovine coronavirus (BCoV), NP_742142; Human coronavirus OC43 (OC43), AAT84359. Numbering was made using SARF as a reference.
(TIF)

Figure S2 Binding determinants in the SAM/SAH/Sinfungin binding site. A) LIGPLOT diagram (www.ebi.ac.uk/thornton-srv/software/LIGPLOT/) of the SAH ligand molecule interacting with the nsp16 binding site. Ligand bonds are in purple, neighbor residue (non-ligand) bonds are in light brown, hydrogen bonds are green dashed lines. Ligand atoms surrounded by a yellow circle are highly accessible. Non-ligand residues in hydrophobic contacts with the ligand are presented by red semi-circles with radiating spokes. B) Superimposition of SAH and Sinefungin molecules. The nsp16 residues are green sticks, the SAH is represented in red sticks, and Sinefungin and water molecules are in sticks and spheres as in Fig. 3, respectively.
(TIF)

Figure S3 Primary and secondary structure elements of nsp16. Helices and sheets are colored according to rainbow colors from N- to C-terminus as in Fig. 1. Patches I to IV are boxed in grey, labeled above the sequences, and correspond to residues represented in Figs. 6A and B.
(TIF)

Figure S4 Nsp10 interface comparison between the nsp10/nsp16 complex and nsp10 homo multimer. A) For the corresponding nsp10/nsp16 interface nsp10 (cyan) interacts with 2 monomers (green and orange). Left: Cartoon representation of 3 nsp10 monomer part of the dodecamer structure (PDB: 2G9T). Right: Cartoon representation of the nsp10(cyan)-nsp16(dark red) complex. Nsp10 molecules in cyan are in the same orientation. B) Nsp10 sequence is presented with above the corresponding secondary structure elements. Below dark red colored dots indicate residues involved in the interaction between nsp10 and nsp16 while green and orange dots indicate residues involved in the homomultimer in the dodecamer complex.
(TIF)

Table S1 Effect of alanine mutations in nsp10 interface residues. Residues were identified using PISA (http://www.ebi.ac.uk/msd-srv/prot_int/pi_link.html). Bold on grey background: strictly conserved residues amongst coronaviruses; Bold on white background: conserved a.a. (>70%) amongst coronaviruses (see Fig. S1). The % of Bioluminescence Resonance energy Transfer (BRET) signal was previously reported[18]. The interaction of each nsp10 or nsp16 mutant was determined using strep-tactin pull-down experiments of strep-tagged nsp10 co-expressed with nsp16 followed by SDS-PAGE analysis, and quantitation (see Methods). The interaction of wild-type nsp10 with wild-type nsp16 was normalized to 100%. The % of MTase activity was determined using filter binding assays (see Methods) relative to wild-type.
(DOC)

Table S2 Effect of alanine mutations in nsp16 interface residues. Residues were identified using PISA (http://www.ebi.ac.uk/msd-srv/prot_int/pi_link.html). Same legend as Table S1 except that no BRET experiments were performed.
(DOC)
