# Supplementary figures and images for: Crystal Structure and Functional Analysis of the SARS-Coronavirus RNA Cap 2′-O-Methyltransferase nsp10/nsp16 Complex
Source: PLoS Pathog. 2011 May 26;7(5):e1002059. doi: 10.1371/journal.ppat.1002059 (PMC3102710; doi:10.1371/journal.ppat.1002059)

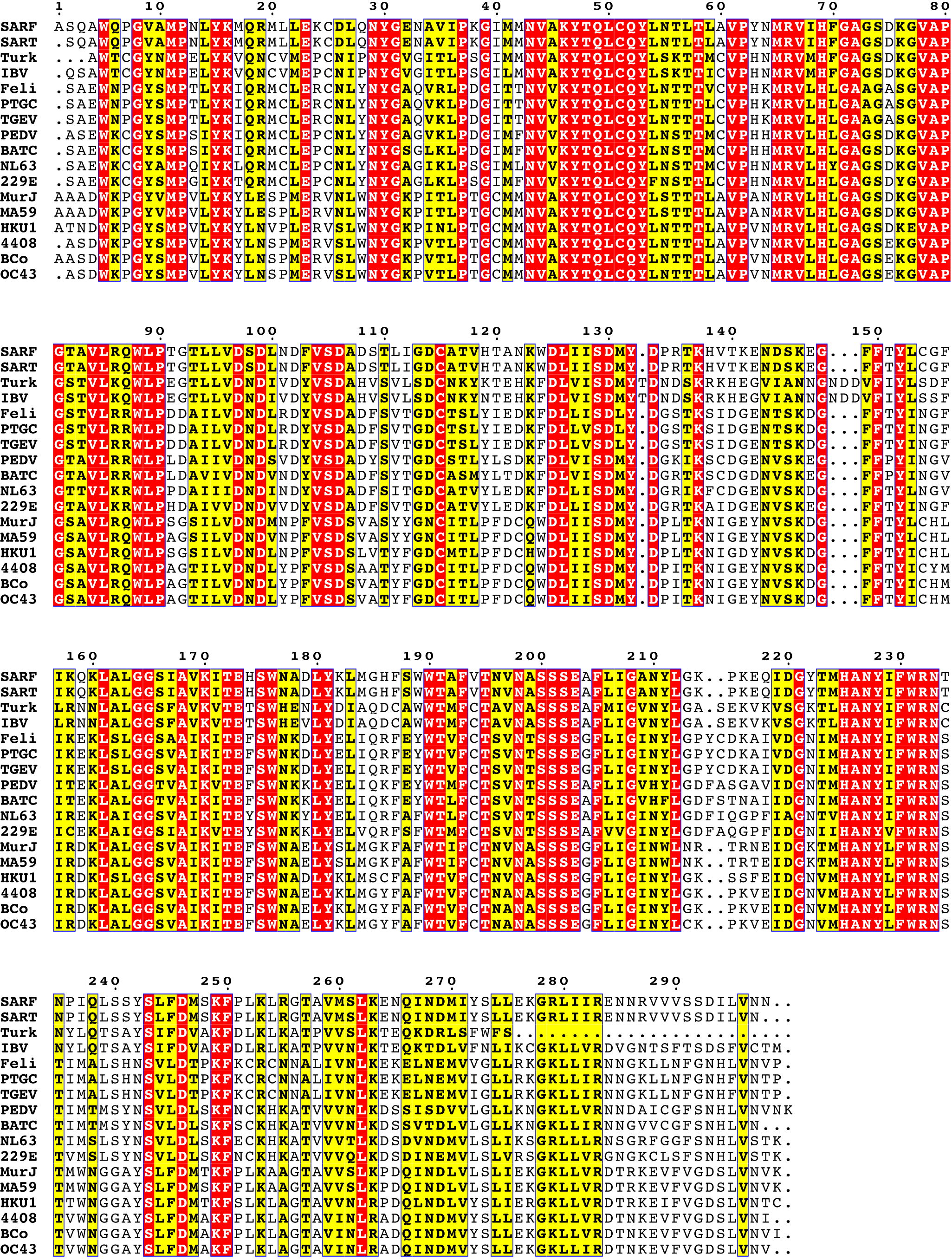

Supplement: Figure S1 — Sequence alignment and amino acid conservation in coronavirus nsp16. The alignment of coronavirus nsp16 sequences was generated with Muscle program (http://www.ebi.ac.uk/Tools/msa/muscle/), and the resulting alignment converted using the ESPript program, (http://espript.ibcp.fr/ESPript/cgi-bin/ESPript.cgi). Residues that are conserved in all or >70% sequences are boxed in red and yellow, respectively. National Center for Biotechnology Information (NCBI) accession numbers for replicase polyprotein sequences that include nsp16 are as follows: Severe acute respiratory syndrome virus SARS-CoV Frankfurt isolate (SARF), Severe acute respiratory syndrome virus SARS-CoV Tor2 isolate (SART), NP_828873.2; Turkey coronavirus (Turk), YP_001941189; Infectious Bronchitis Virus (IBV), NP_066134; Feline Coronavirus (Feli), YP_239426;Porcine Transmissible Gastroenteritis Coronavirus (PTGC), P18457; Transmissible Gastroenteritis coronavirus (TGEV), NP_840013; Porcine epidemic diarrhea virus CV777 (PEDV), NP_839969; Bat coronavirus 512/2005 (BATC), YP_001351683; Human coronavirus NL63 (NL63), AAS58176; Human coronavirus 229E (229E), NP_073549; Murine hepatitis virus strain JHM (MurJ), YP_209243; Mouse Hepatitis virus strain A59 (MA59), NP_740613; Human coronavirus HKU1 genotype A (HKU1), YP_460023; Human enteric coronavirus 4408 (4408), ACJ35483; Bovine coronavirus (BCoV), NP_742142; Human coronavirus OC43 (OC43), AAT84359. Numbering was made using SARF as a reference. (TIF) [file ppat.1002059.s001.tif]

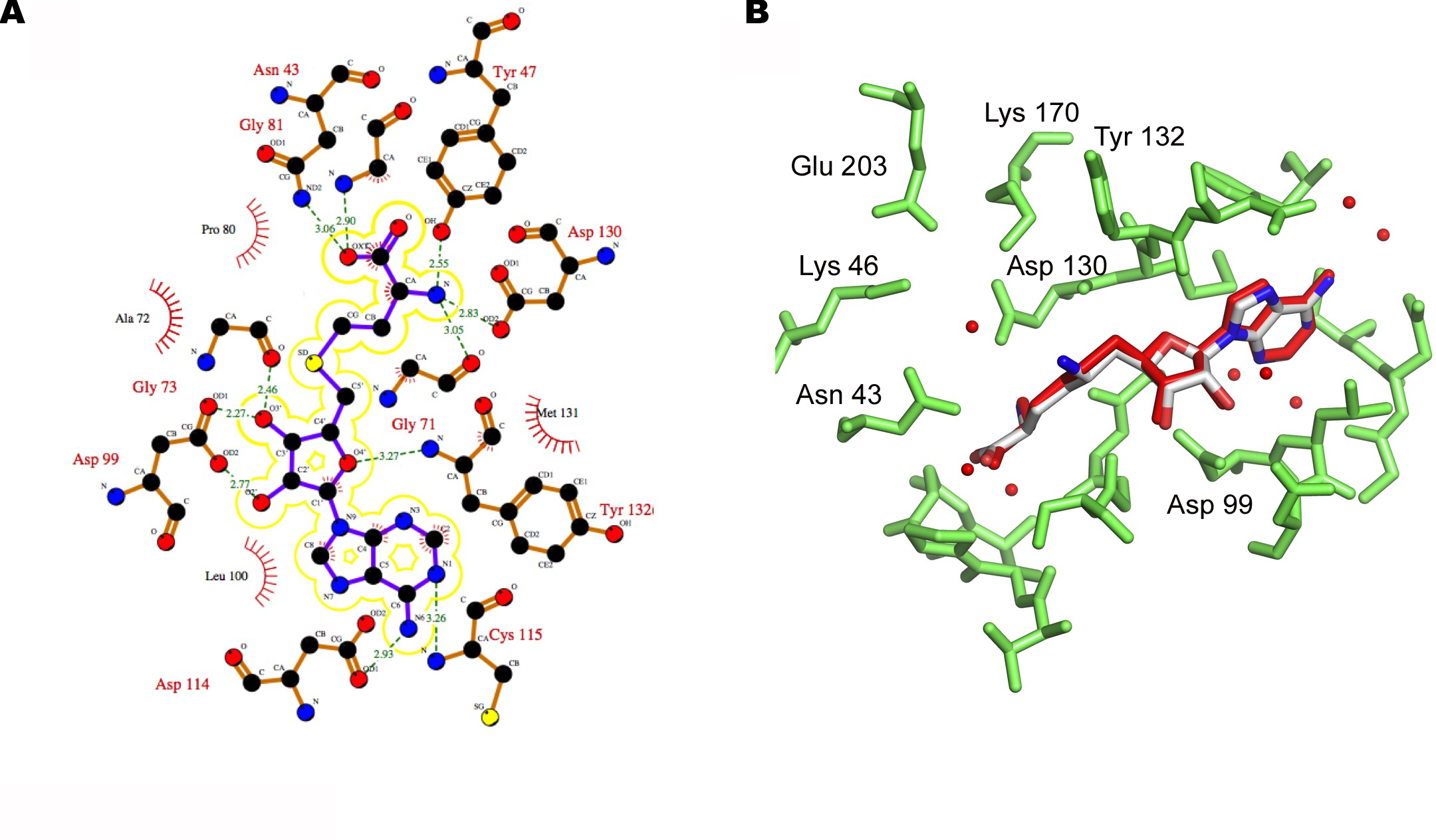

Supplement: Figure S2 — Binding determinants in the SAM/SAH/Sinfungin binding site. A) LIGPLOT diagram (www.ebi.ac.uk/thornton-srv/software/LIGPLOT/) of the SAH ligand molecule interacting with the nsp16 binding site. Ligand bonds are in purple, neighbor residue (non-ligand) bonds are in light brown, hydrogen bonds are green dashed lines. Ligand atoms surrounded by a yellow circle are highly accessible. Non-ligand residues in hydrophobic contacts with the ligand are presented by red semi-circles with radiating spokes. B) Superimposition of SAH and Sinefungin molecules. The nsp16 residues are green sticks, the SAH is represented in red sticks, and Sinefungin and water molecules are in sticks and spheres as in Fig. 3, respectively. (TIF) [file ppat.1002059.s002.tif]

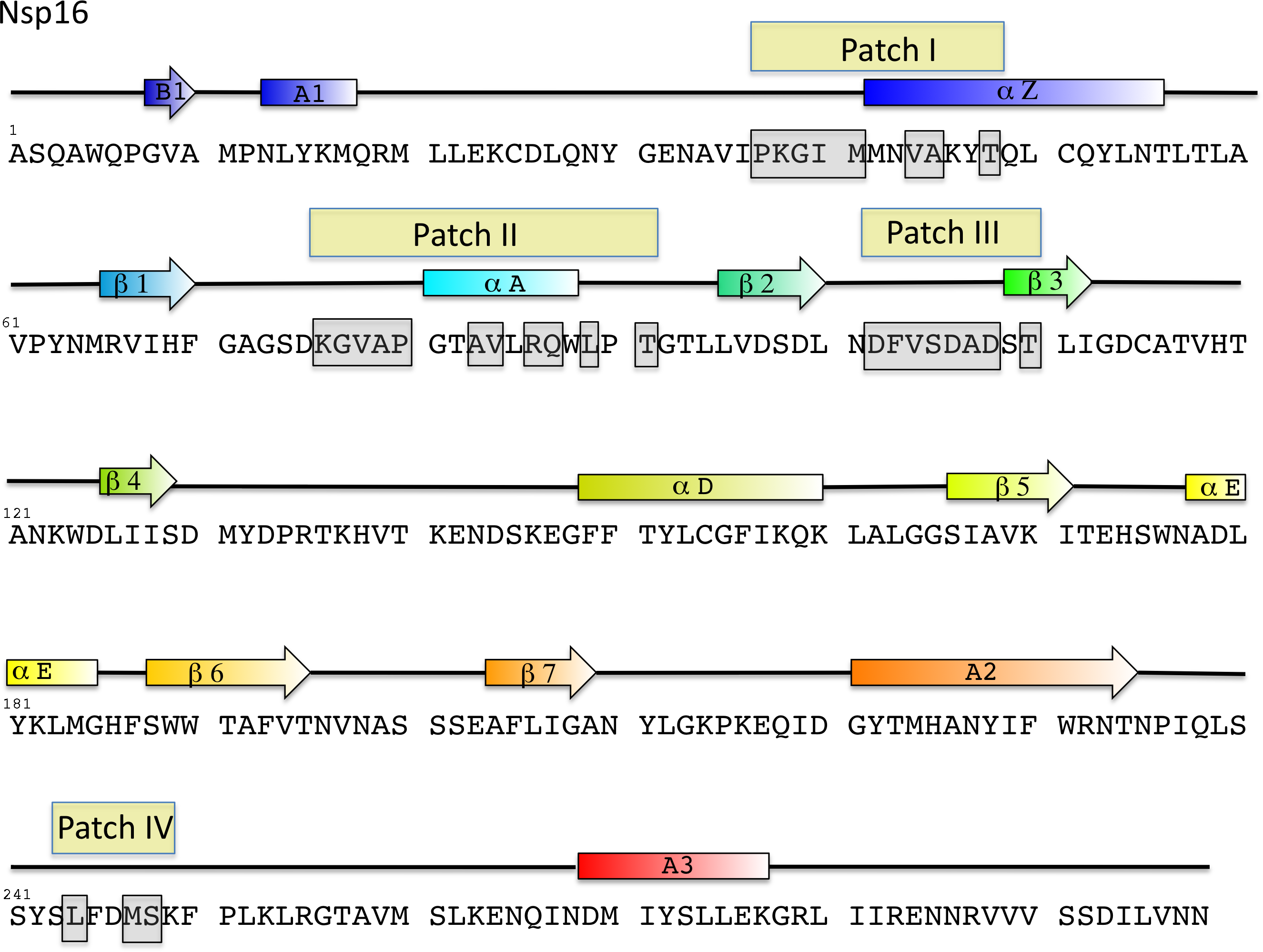

Supplement: Figure S3 — Primary and secondary structure elements of nsp16. Helices and sheets are colored according to rainbow colors from N- to C-terminus as in Fig. 1. Patches I to IV are boxed in grey, labeled above the sequences, and correspond to residues represented in Figs. 6A and B. (TIF) [file ppat.1002059.s003.tif]

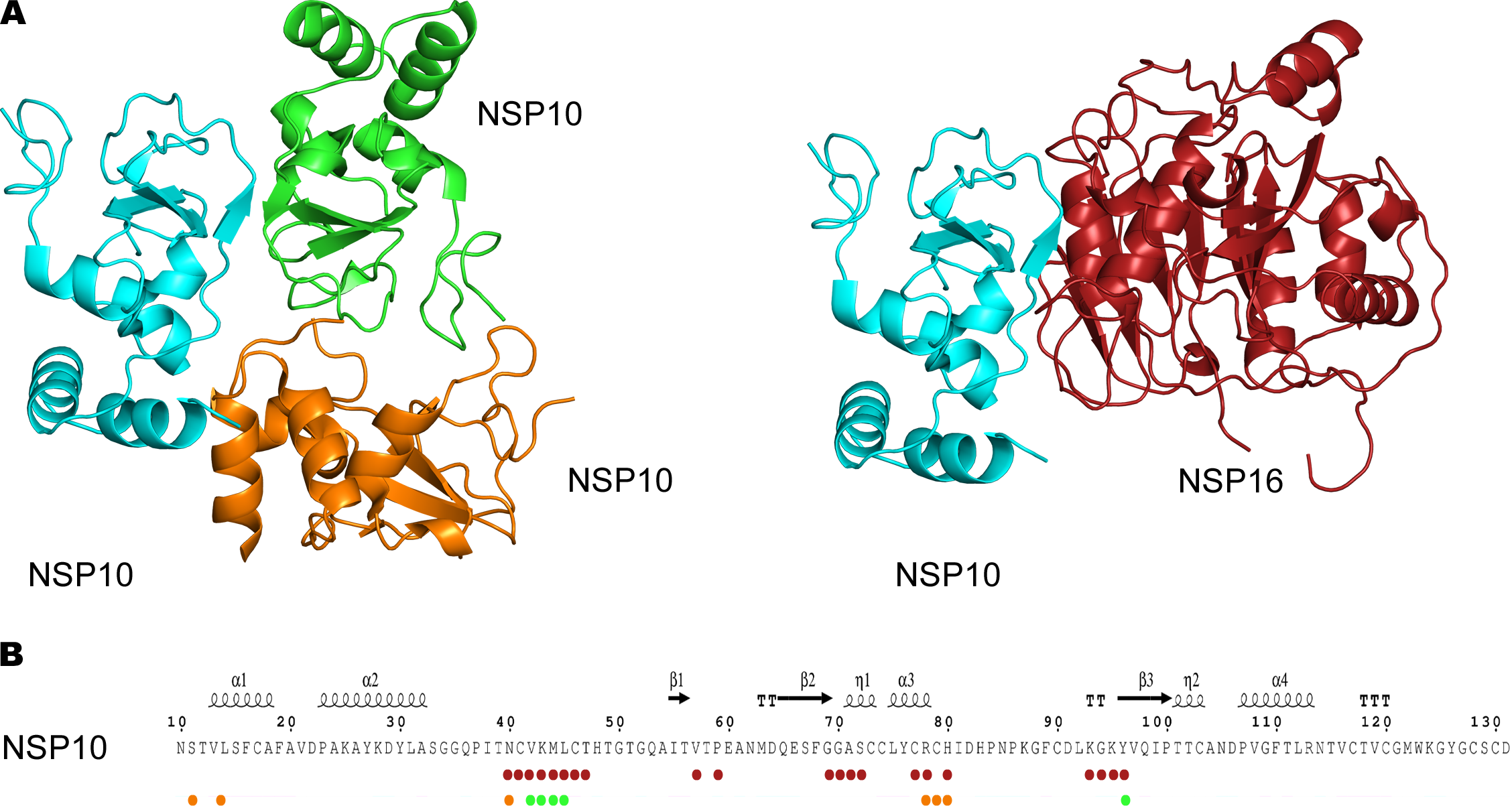

Supplement: Figure S4 — Nsp10 interface comparison between the nsp10/nsp16 complex and nsp10 homo multimer. A) For the corresponding nsp10/nsp16 interface nsp10 (cyan) interacts with 2 monomers (green and orange). Left: Cartoon representation of 3 nsp10 monomer part of the dodecamer structure (PDB: 2G9T). Right: Cartoon representation of the nsp10(cyan)-nsp16(dark red) complex. Nsp10 molecules in cyan are in the same orientation. B) Nsp10 sequence is presented with above the corresponding secondary structure elements. Below dark red colored dots indicate residues involved in the interaction between nsp10 and nsp16 while green and orange dots indicate residues involved in the homomultimer in the dodecamer complex. (TIF) [file ppat.1002059.s004.tif]
