# Supplementary material for: Crystal Structure and Functional Analysis of the SARS-Coronavirus RNA Cap 2′-O-Methyltransferase nsp10/nsp16 Complex
Source: PLoS Pathog. 2011 May 26;7(5):e1002059. doi: 10.1371/journal.ppat.1002059 (PMC3102710; doi:10.1371/journal.ppat.1002059)
Supplement: Table S1 — Effect of alanine mutations in nsp10 interface residues. Residues were identified using PISA (http://www.ebi.ac.uk/msd-srv/prot_int/pi_link.html). Bold on grey background: strictly conserved residues amongst coronaviruses; Bold on white background: conserved a.a. (>70%) amongst coronaviruses (see Fig. S1). The % of Bioluminescence Resonance energy Transfer (BRET) signal was previously reported[18]. The interaction of each nsp10 or nsp16 mutant was determined using strep-tactin pull-down experiments of strep-tagged nsp10 co-expressed with nsp16 followed by SDS-PAGE analysis, and quantitation (see Methods). The interaction of wild-type nsp10 with wild-type nsp16 was normalized to 100%. The % of MTase activity was determined using filter binding assays (see Methods) relative to wild-type. (DOC) [file ppat.1002059.s005.doc]

**Table S1**

| # Nsp10 | Patch | % of BRET signal | % of interaction | % of MTase  Activity |
| --- | --- | --- | --- | --- |
| Asn10 | A | - | - | - |
| **Asn 40** | A | - | 64 | 35 |
| **Cys 41** | A | - | - | - |
| **Val 42** | A | 14 | 1 | 1 |
| **Lys 43** | A | - | - | - |
| **Met 44** | A | 8 | 0 | 0 |
| **Leu 45** | A | - | 6 | 0 |
| **Cys 46** | A | - | - | - |
| **Thr 47** | A | - | 66 | 10 |
| **Val 57** | B | 98 | - | 96 |
| Thr 58 | B | - | 93 | 112 |
| **Pro 59** | B | - | - | - |
| **Gly 69** | C | - | 92 | - |
| **Gly 70** | C | 31 | 51 | 32 |
| **Ala 71** | C | - | - | - |
| **Ser 72** | C | 59 | 6 | 21 |
| **Cys 77** | D | - | - | - |
| **Arg 78** | D | 8 | 10 | 2 |
| **His 80** | D | - | 85 | 38 |
| **Lys 93** | E | 35 | 54 | 9 |
| **Gly 94** | E | 58 | 80 | 84 |
| **Lys 95** | E | 82 | 87 | 68 |
| **Tyr 96** | E | 30 | 6 | 14 |
