# Supplementary material for: Crystal Structure and Functional Analysis of the SARS-Coronavirus RNA Cap 2′-O-Methyltransferase nsp10/nsp16 Complex
Source: PLoS Pathog. 2011 May 26;7(5):e1002059. doi: 10.1371/journal.ppat.1002059 (PMC3102710; doi:10.1371/journal.ppat.1002059)
Supplement: Table S2 — Effect of alanine mutations in nsp16 interface residues. Residues were identified using PISA (http://www.ebi.ac.uk/msd-srv/prot_int/pi_link.html). Same legend as Table S1 except that no BRET experiments were performed. (DOC) [file ppat.1002059.s006.doc]

**Table S2**

| # Nsp16 | Patch | % of interaction | % of MTase  Activity |
| --- | --- | --- | --- |
| **Pro 37** | I | - | - |
| Lys 38 | I | - | - |
| **Gly 39** | I | - | - |
| Ile 40 | I | 84.2 | 7.5 |
| Met 41 | I | 122.3 | 3.8 |
| **Val 44** | I | 113.0 | 0.7 |
| **Ala 45** | I | - | - |
| **Thr 48** | I | 122.6 | 20.1 |
| Lys 76 | II | - | - |
| **Gly 77** | II | - | - |
| **Val 78** | II | 2.6 | 0.2 |
| **Ala 79** | II | - | - |
| **Pro 80** | II | - | - |
| **Ala 83** | II | - | - |
| **Val 84** | II | - | - |
| **Arg 86** | II | 60.8 | 0.05 |
| **Gln 87** | II | 114.0 | 61.6 |
| **Leu 89** | II | - | - |
| Thr 91 | II | - | - |
| Asp 102 | III | - | - |
| **Phe 103** | III | - | - |
| **Val 104** | III | 5.6 | 4.3 |
| **Ser 105** | III | - | - |
| **Asp 106** | III | 101.1 | 38.4 |
| **Ala 107** | III | - | - |
| Asp 108 | III | - | - |
| **Thr 110** | III | - | - |
| **Leu 244** | IV | 30 | 0 |
| **Met 247** | IV | 4 | 0 |
| Ser 248 | IV | - | - |
